# Research Progress on the Preparation Technology of Spherical Alloy Powders for Laser Additive Manufacturing

**DOI:** 10.3390/ma18143385

**Published:** 2025-07-18

**Authors:** Sen Zhang, Kuaikuai Guo, Yongquan Qing, Changsheng Liu

**Affiliations:** 1 School of Materials Science and Engineering, Northeastern University, Shenyang 110819, China; tskxy2014@163.com (S.Z.); neukuai@163.com (K.G.); qingyq@mail.neu.edu.cn (Y.Q.); 2 Taishan Science and Technology Research Institute (Taian Innovation and Development Research Institute), Tai’an 271000, China; 3 Institute of Metal Research, Chinese Academy of Sciences, Shenyang 110016, China

**Keywords:** laser additive manufacturing, alloy powder, gas atomization, plasma atomization, centrifugal atomization

## Abstract

Spherical powder materials are essential raw materials for manufacturing processes such as metal additive manufacturing and powder metallurgy. They possess characteristics that are key factors influencing the performance of additive manufacturing. This paper introduces the fundamental principles and characteristics of laser additive manufacturing technology and analyzes the technical principles, advantages, and disadvantages of three alloy powder preparation methods: gas atomization, centrifugal atomization, and plasma atomization. It further elucidates the influence of process parameters of these three powder preparation techniques on the characteristics of alloy powders. Finally, the development trends in alloy powder preparation for laser additive manufacturing are projected.

## 1. Introduction

Additive manufacturing (AM) refers to a low-loss laminated processing technology that utilizes computer software to establish a three-dimensional model of a component and processes and shapes discrete materials (such as powder, liquid, wire, etc.) through a specific printing technique via layer-by-layer melting and accumulation [1,2]. Additive manufacturing technology has advantages such as a high overall material utilization rate, few processes, high design freedom, the ability to manufacture complex-structured parts, easy realization of intelligence, and high efficiency. It has been widely applied in fields such as aerospace, automotive manufacturing, petrochemicals, metallurgy, and railway locomotives [3]. Laser additive manufacturing technology, as one type of additive manufacturing technology, not only can enhance the wear resistance, corrosion resistance, and thermal stability of mechanical parts, but also has significant advantages such as high efficiency, rapidity, and suitability for processing complex parts. It is more suitable for manufacturing components with complex geometries, high flexibility, and high automation [4,5].

Alloy powder materials are crucial factors influencing the performance of laser additive manufacturing. Properties such as the chemical composition, sphericity, porosity, fluidity, and particle size distribution of the powder have a direct impact on the microstructure and properties of the additive manufacturing [6]. Metal powders suitable for additive manufacturing technology must also meet the criteria of low oxygen content, high sphericity, good fluidity, and high purity [7,8]. Alloy powders primarily include titanium alloy powders, high-strength steels, and superalloys. With the rapid advancement of additive manufacturing technology, there is a significant demand for powder metallurgy both domestically and internationally. The performance of alloy powders directly affects the forming performance of 3D printed titanium alloy parts. The preparation technology and processes of titanium powder for 3D printing have become focal points for development and research both at home and abroad.

As an emerging branch of technology in the field of additive manufacturing, biological 3D printing combines 3D printing with biomaterials, utilizing active molecules and cells as basic building units to produce bionic products, tissues, and even organs through precise control of the assembly process. Metal materials such as cobalt–chromium alloys, nickel alloys, and titanium alloys are widely used in biological 3D printing due to their excellent biocompatibility, corrosion resistance, and high strength. In the aerospace industry, 3D printing technology offers higher manufacturing precision and is not limited by the shape and structure of components, making it suitable for the processing and manufacturing of high-hardness, high-melting-point superalloys and titanium alloys.

The research team from Northeastern University has conducted extensive studies on the preparation of alloy powders by atomization, the influence and mechanism of process parameters on the performance and quality of coatings during cladding, etc. TC4 titanium alloy powder was prepared using electrode vacuum induction gas atomization technology. A comprehensive electrode induction melting gas atomization (EIGA) system, featuring high-vacuum, induction melting, and gas atomization functions, was designed and integrated. Process parameters were optimized to prepare Ti-6Al-4V alloy powder. The microstructure and mechanical properties of titanium alloy parts formed by LMD technology were characterized and tested. The effects of melting power, atomization gas pressure, and other factors on the surface morphology, particle size distribution, and fluidity of TC4 titanium alloy powder were investigated. This paper reviews the relevant research status from aspects such as laser additive manufacturing technology, alloy powder preparation technology, and the influence of process parameters on the characteristics of alloy powders and looks forward to its future development.

## 2. The Principle and Characteristics of Laser Additive Manufacturing Technology

**Figure 1:**
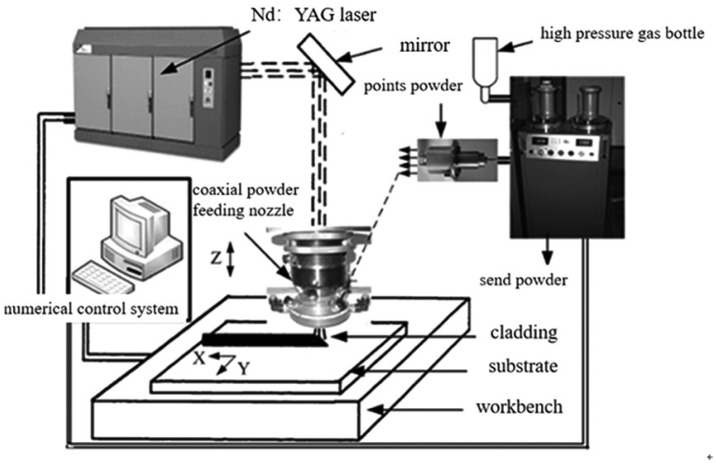
Schematic diagram of laser additive manufacturing technology. Reprinted from Ref. [9].

Laser additive manufacturing technology is a process in which a laser beam serves as the heat source. The coating material is placed on the surface of the substrate to be coated in various ways. The laser irradiation causes a thin layer on the surface of the substrate and the coating material to melt simultaneously. After rapid solidification, a surface layer with an extremely low dilution ratio and metallurgical bonding with the substrate material is formed [9]. The technical principle is illustrated in Figure 1. Firstly, a three-dimensional CAD model of the part to be fabricated is created by a computer. Then, the CAD model is sliced into thin layers of a certain thickness, transforming the complex three-dimensional structure of the part into a series of two-dimensional planar structures. Next, the scanning trajectory for each two-dimensional contour is designed, and the processed data is transmitted to the numerical control system to generate numerical control codes. Finally, under the control of a computer program, the powder material is deposited layer by layer and row by row along the preset path using the laser cladding method, ultimately forming a three-dimensional solid part or a semi-finished part that requires only minimal processing [10]. The most representative laser additive manufacturing technologies are selective laser melting (SLM), characterized by powder bed technology, and laser metal deposition (LMD), characterized by simultaneous powder feeding [11].

According to the shape of each cross-section of the component, SLM builds up the component layer by layer. During the printing process, SLM typically employs a laser spot of micrometer scale, resulting in a smaller and more controllable melt pool. This enables the production of high-density, high-precision, and complex-shaped components. The printed components can meet usage requirements after simple treatments such as grinding or sandblasting [12]. Due to the need for layer-by-layer powder spreading in SLM, the thickness of each powder layer is usually only a few tens of micrometers, which significantly limits the printing speed. Especially when manufacturing large-sized components, the forming cycle will increase significantly. In SLM, only specific areas of the powder are melted, and although the unmelted powder can be recycled and reused, the incorporation of splashed powder can cause issues such as deterioration of powder performance. Selective laser melting forming has high requirements for powder quality, and usually, metal powders with a small particle size and narrow particle size distribution are selected. A suitable powder particle size is 15–53 μm, with a sphericity of over 98%, minimal satellite powder, an oxygen content of less than 0.01%, high bulk density, etc. [13]. The finer the powder particles, the smaller the gap between particles and the denser the powder layer, which is conducive to obtaining high-quality metal parts with high sintering strength. If the selected powder is coarser and has a wider particle size distribution, the porosity, strength, surface roughness, etc., of the formed parts will increase significantly.

Laser metal deposition (LMD) technology employs a method where the laser, powder, and shielding gas move synchronously. Its principle is to transport the metal raw material to the laser action area through a nozzle. Under the local shielding atmosphere ejected from the nozzle, a high-energy laser beam is used to melt the input raw material and deposit it layer by layer for forming [14]. Compared with selective laser melting (SLM), LMD is not restricted by the size of the powder bed, enabling the manufacturing and repair of larger components and demonstrating higher flexibility and broader application potential. LMD typically uses a millimeter-sized laser spot, resulting in a wider and deeper molten pool. Therefore, powders with larger particle sizes can be used, and even larger-sized welding wires can be directly adopted as raw materials, thereby achieving the goal of improving deposition efficiency. However, the way LMD introduces flowing shielding gas around the molten pool increases the probability of the molten pool capturing gas, making it easier to form pore defects, which in turn affect the forming quality and density of the components [15]. Laser metal deposition (LMD) has a relatively wide adaptability to powder particle size and is suitable for printing large-sized parts with large machining allowances. The powder application range spans from fine powders of several tens of microns to coarse powders of several hundreds of microns. Usually, powders with a particle size of 53–150 μm are used as consumables. The powders are required to have good sphericity (greater than 85%), low oxygen content (less than 0.03%), and good uniformity.

Laser additive manufacturing (LAM) technology, with its characteristics of concentrated heat input, rapid cooling rate, extremely low dilution rate, fine-grained microstructure in cladding layers, superior performance, precise control over process parameters, capability for accurate repair of specific component areas, excellent operability, high efficiency, and minimal environmental pollution [16], has emerged as a cutting-edge additive manufacturing technology prioritized for development worldwide. It is extensively utilized in the repair and manufacturing of high-precision, high-added-value components with stringent performance requirements. The shape of particles and the surface state of powders can affect the apparent density and tapped density of the powders. The LMD process has higher requirements for powder fluidity and powder surface roughness, while the SLM process has higher requirements for apparent density.

## 3. Powder Metallurgy Preparation Technology

The atomization method is a mature and widely used technique for producing high-performance spherical metal and alloy powders. Powders prepared by atomization account for approximately 80% of the global powder production today. Commonly used technologies for manufacturing spherical powders mainly include gas atomization, centrifugal atomization, plasma atomization, and other related methods [17]. The standardization and certification of powders in additive manufacturing are critical factors in assessing the quality of alloy powders, especially in the medical and aerospace fields. The International Organization for Standardization (ISO) has published the ISO/ASTM 52938-1:2025 standard [18], which stipulates safety requirements for metal additive manufacturing equipment, particularly for machines using metal powder beds. China has issued a series of national standards in the field of additive manufacturing, covering materials, equipment, design, and other aspects. For example, in 2024, 11 national standards were published, with a significant increase in standardization related to metal powders, including materials such as aluminum alloys, die steels, magnesium alloys, and nickel–titanium alloys. In addition, China has formulated a standard for high-entropy alloy powders (GB/T 42787-2023 [19]), filling the gap in the national standard system for special high-entropy alloy powders used in additive manufacturing in China.

### 3.1. Gas Atomization Technology (GA)

**Figure 2:**
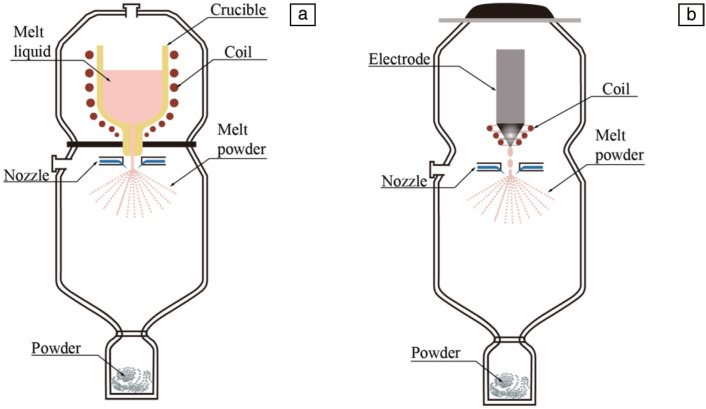
Schematic of the gas atomization process for the production of AM powders. Reprinted from Ref. [23]. (**a**) VIGA; (**b**) EIGA.

Gas atomization pulverization technology originated in the 1820s and was initially used for the preparation of non-ferrous metal powder. In the late 1970s and early 1980s, computer technology and modern control technology were integrated into the industrial production of gas atomization pulverization, leading to significant advancements in the research on the mechanism of gas atomization [20]. The principle of gas atomization pulverization technology involves converting the kinetic energy of the gas into the surface energy of the melt by acting on the melt flow with high-speed air flow and then forming fine droplets that are solidified into powder particles. Currently, typical gas atomization technologies are vacuum induction melting gas atomization (VIGA) [21] and electrode induction melting gas atomization (EIGA) [22]. Vacuum induction melting gas atomization technology completes the melting and degassing process of alloy raw materials in the crucible under vacuum conditions, and then the molten metal flows through the diversion tube to form a stable liquid column. The liquid flow meets the high-pressure gas under the control of the nozzle and is broken into fine metal droplets. The molten metal droplets are constantly cooled and spheroidized by high-speed high-pressure gas in the atomization chamber. Finally, they solidify into a spherical powder [23], as shown in Figure 2a. Electrode induction argon atomization is an improvement over vacuum induction melting gas atomization. The technology uses the shaped alloy rod material as the electrode, and the alloy rod is heated and melted through the high-frequency induction coil in the vacuum chamber to form a continuous and controllable molten metal flow with a fixed diameter. The alloy liquid flow flows into the atomization chamber under the action of gravity (no diversion tube). Metal droplets are atomized into metal droplets in contact with high-pressure air flow, and then the droplets complete the process of cooling, spheroidization, and solidification in the atomization chamber and finally solidify into spherical powder [24], as shown in Figure 2b.

**Figure 3:**
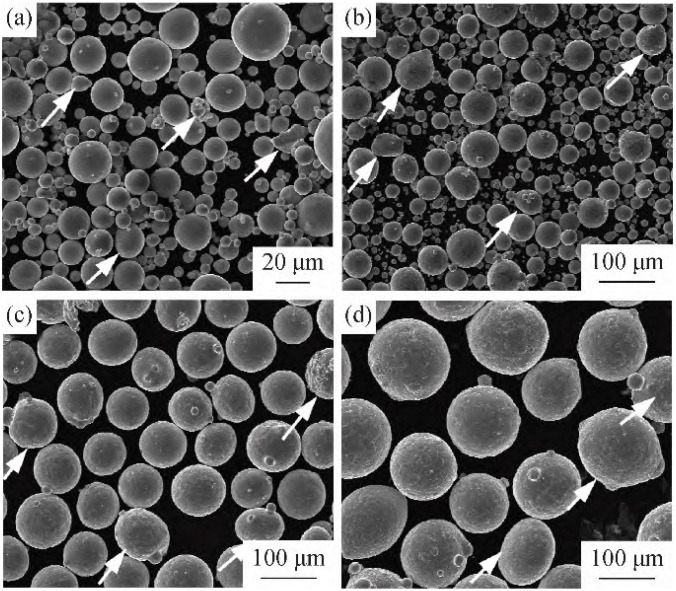
SEM surface morphology of TiAl alloy powders in different particle size intervals: (**a**) ≤45 μm, (**b**) 45~75 μm, (**c**) 75~100 μm, (**d**) ≥100 μm. Reprinted from Ref. [27].

In the process of powder preparation by GA, the factors affecting atomization efficiency and powder performance include atomization pressure, atomization equipment, atomization gas, etc. Zhao Shao Yang [25] prepared TiSi_2_ alloy powders via vacuum induction melting gas atomization (VIGA). Comprehensive characterization was conducted on the physical properties, surface morphology, and phase composition of the as-produced powders. The results indicated that the powder exhibited a highly spherical microstructure with partial satellite particles. The particle size distribution ranged from 15 to 150 μm, achieving a yield rate of >95% for particles <150 μm, demonstrating excellent fine-powder recovery. The oxygen content of the powders was measured at 0.026–0.030% (mass fraction). The authors attributed the slightly higher oxygen content in smaller particles to their larger specific surface area and enhanced reactivity, which facilitated oxygen adsorption during storage. The overall oxygen content of ≤0.03% validated the effectiveness of the VIGA process, particularly the clean melting advantage of water-cooled copper crucibles and the protective atmosphere of high-purity argon during atomization. Zhang Guo qing [26] used the vacuum induction atomization (VIGA) method to carry out research on the preparation and application of 3D printed high-temperature alloy powder materials. The yield of the prepared high-alloy fine powder (≤53 μm) was greater than 70%, the sphericity was ≥0.90, and the O content of the powder was ≤0.02%. The industrialization of 3D printed high-temperature alloy powder was basically realized. Nickel-based alloy powders such as GH416, GH3536, GH/PM625, and GH/PM625M are currently used in the 3D printing of related parts of aeroengines. Wang Hu [27] prepared Ti-48Al-2Cr-2Nb intermetallic compound powder by electro-induction gas atomization (EIG), and the results showed that the powder size was mainly distributed between 15 and 105 μm, and the powder exhibited normal distribution characteristics, with an average O content of 0.092%. The shape of the powder was mainly spherical or near-spherical, but a small amount of abnormally shaped powder also appeared (as indicated by the arrows in the figure), including satellite powder, ellipsoidal powder, wrapped powder, and twin powder. SEM images of TiAl alloy powders in different size intervals are shown in Figure 3.

Gas atomization technology can effectively prevent the oxidation and burning of alloying elements, improve the solid solution of alloying elements and the shape, size, and distribution of the second phase, reduce segregation, and refine grains. It has the advantages of a low cost, wide application range, and high fine-powder harvest rate [28,29], Both EIGA and VIGA atomization powder production technologies can provide metal powder with stability and repeatability at present, and compared to VIGA, IGA has higher stability because there is no step of crucible melting, and the liquid droplets are directly atomized into powder after induction melting [30]. However, there are also disadvantages such as the composition segregation of the melting electrode leading to the non-uniformity of the powder composition, the rough surface of the powder, the conjoined satellite balls, and the defects of some powder particles [31]. The main manufacturers of EIGA equipment are AVIC MET (Beijing, China), Zhuzhou Hanhe (Zhuzhou, Hunan, China), ALD (Hanau, Germany), etc.

### 3.2. Centrifugal Atomization Technology (CA)

**Figure 4:**
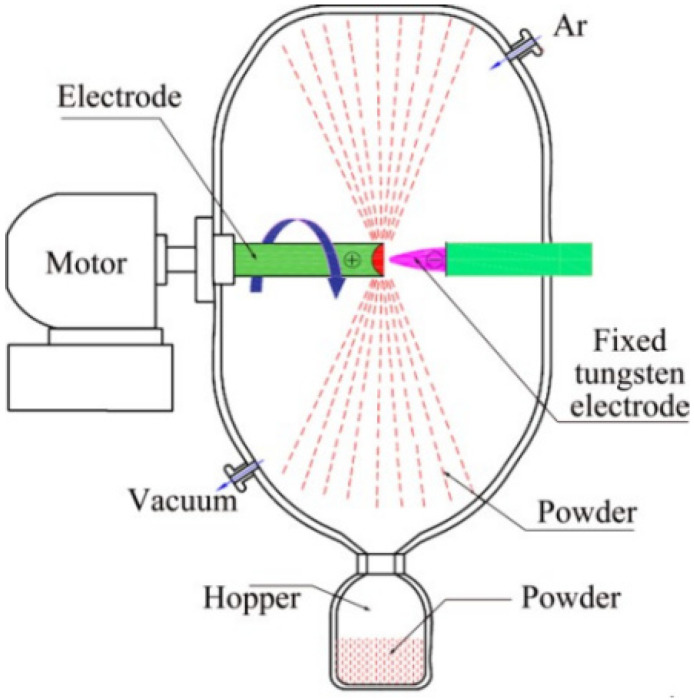
Schematic of plasma rotating electrode process (PREP). Reprinted with permission from Ref. [33]. Copyright 2017 Elsevier.

Centrifugal atomization (CA) is a technology that employs centrifugal force to fragment the melt, dispersing it into droplets that solidify into spherical powder during flight. The plasma rotating electrode process (PREP) is the most prevalent form of centrifugal atomization used for producing high-purity spherical titanium powder [32]. The technical principle involves processing the parent alloy into an electrode rod, which is then continuously melted by a plasma arc serving as the heat source. The molten metal droplets at the end of the alloy rod are expelled by centrifugal force and further fragmented by shear stress as they collide with the inert gas (argon) in the atomization chamber. Subsequently, the droplets rapidly cool and solidify into spherical powder, driven by surface tension [33], as depicted in Figure 4.

**Figure 5:**
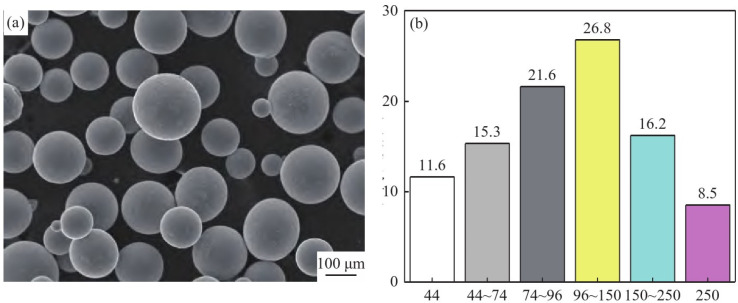
Microstructure (**a**) and particle size distribution (**b**) of Ti185 alloy powders prepared by PREP. Reprinted from Ref. [38].

In the process of PREP powder, the composition of the parent material plays a decisive role in the composition of the powder prepared finally and ultimately affects the performance of the product. In the process of melting the parent, precise chemical composition control is essential. Tang Huiping [34] discovered that the pores in the plasma rotating electrode powder were caused by low surface tension, making it easier to produce pore defects within coarse-particle-size powder, that porosity and other defects can significantly reduce the material’s density and mechanical strength, and that when the component is subjected to external force, stress concentration occurs around porosity, resulting in local stresses far higher than the average stress level. When the porosity rate in metal components reaches a certain level, their tensile strength and yield strength may be reduced by 20–50%, and the toughness will also decrease significantly. Yang Xin [35] utilized the plasma beam rotating electrode method to produce Ti-47Al-2Cr-2Nb spherical powder with a particle size distribution ranging from 30 to 250 μm, an average particle size (d50) of 85 μm, a bulk density of 2.65 g/cm^3^, a sphericity rate of 99.6%, and an average oxygen content of 0.05%. As the powder particle size decreased, the oxygen content increased sharply but did not surpass 0.10%. Xie Zhonghao [36] prepared high-sphericality, low-oxygen-content TaNbTiZr refractory high-entropy alloy powder using the PREP method, resulting in good compositional uniformity and a low impurity content, with the oxygen mass fraction of only 0.0777%; the high-quality spherical powder of TaNbTiZr refractory high-entropy alloy prepared by the plasma rotating electrode atomization method (PREP) was used as a raw material, and the structure and mechanical properties of TaNbTiZr refractory high-entropy alloy prepared by electron beam selective melting (EBM) were investigated. The results show that the NbTiZr refractory high-entropy alloy shows a fine equiaxed grain structure and has excellent mechanical properties [37]. Li Zengfeng [38] employed the plasma rotating electrode atomization technique to prepare spherical Ti185 alloy powder. The resulting alloy powder was entirely composed of the β phase, with particles exhibiting high sphericity and a lack of irregular or satellite powder. The powder particle size distribution was broad, primarily between 44 and 150 μm, with a maximum size reaching 250 μm. The microstructure and particle size distribution of the Ti185 alloy powder are depicted in Figure 5. The results indicated that the oxygen content (mass fraction) of the powder was under 0.14%. Powder with a particle size ≤ 44 μm had a smooth surface, and its internal structure was notably refined, with a powder recovery rate of 11.6%. The fluidity of powder ≤ 150 μm was 24.79 s/50 g, the bulk density was 2.79 g/cm^3^, and the vibration density was 2.99 g/cm^3^, meeting the requirements for powder bed 3D printing technology.

The powder obtained by plasma rotating electrode technology has high sphericity and internal density. The powder particles inherit the composition uniformity of the titanium alloy electrode rod after many vacuum smelting cycles, and there is almost no obvious component segregation between the particles. The powder has high sphericity, good fluidity, high purity, and low oxygen content. The average particle size of the prepared alloy powder is 80~120 µm, which can be used for the powder preparation of various component metal materials such as nickel-based superalloy, aluminum alloy, stainless steel, and the like. The main disadvantages are that when the plasma matrix exceeds a certain value, it will cause droplet ashing and solidification to form satellite powder, and it is also limited by the applicability of materials. For example, when high-activity metals (such as titanium, zirconium) are atomized, the molten metal may react with or pollute the electrode of the plasma torch (usually tungsten), affecting the electrode life and powder purity. However, due to the influence of electrode speed and other factors, the powder particle size is mostly greater than 70 µm, the particle size distribution of the prepared powder is narrow, the recovery rate of fine powder is very low, and the powder yield is low, which limits its application scope, and this method has high requirements for equipment [39]. At present, PREP manufacturers include AV Mite, Hunan Topray Technology, Xi’an Sai Long, Russian Electric Machinery Company, etc.

### 3.3. Plasma Atomization Technology (PA)

**Figure 6:**
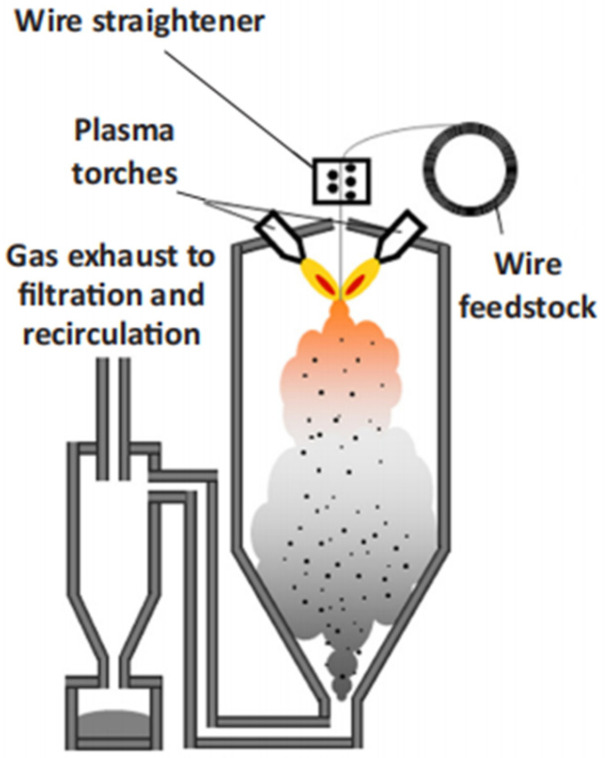
Schematic of the PA process for the production of AM powders. Reprinted from Ref. [41].

Plasma atomization (PA) is a method that uses a plasma torch as a heat source to heat and melt metal wire and simultaneously atomize it to prepare spherical metal powder [40]. The technical principle is to introduce inert gas such as argon into the plasma region; the raw material wire is heated and melted by multiple groups of plasma arcs, and the high power supply generates a high frequency electromagnetic field, which ionizes the gas to form a plasma jet. When the metal self-consuming rod acts as an anode and interacts with the plasma jet, the metal is heated and melted rapidly. Under the impact of the plasma jet, it is broken into a large number of tiny droplets and dispersed at a very fast speed. Under the action of surface tension, it is cooled and solidified into spherical powder [21,41], as shown in Figure 6.

**Figure 7:**
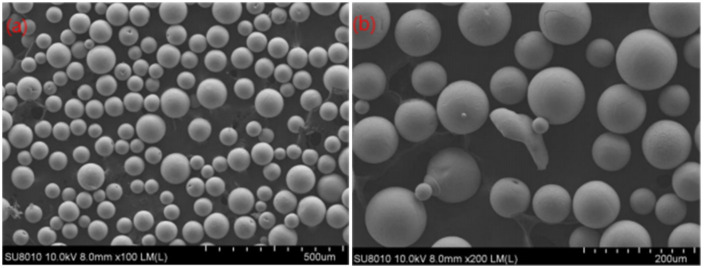
Microscopic morphology of spherical titanium powder prepared by plasma atomization technology; (**a**) 100 times, (**b**) 200 times. Reprinted from Ref. [43].

The powder production enterprises using PA technology in the world are mainly distributed in North America, with representative companies such as AP&C and Pyro Genesis Canada, and have achieved a lot of research progress in recent years. Under the condition that the ratio of gas consumption to raw material mass per unit time is less than 20, the existing technology can prepare metal powder with at least an 80% particle size distribution of 0–106 μm, which can be used in the market of metal molding [17]. Due to patent protection and technology blockades, the research progress of PA technology in China is slow. Zeng Keli [42] found that the particle size of titanium alloy powder is closely related to the plasma jet velocity. The higher the plasma jet velocity, the finer the particle size of the atomized powder. A compressed-expansion nozzle was installed on the anode of the plasma torch. The inlet and outlet sizes of the anode nozzle were 10–12 mm and 6–8 mm, respectively, and the throat size was 4–6 mm. The optimized nozzle structure improved the plasma jet speed and obtained a more refined atomized powder particle size, with the median particle size of the powder decreasing from 70 μm to 40 μm. The prepared titanium alloy TC4 powder, low-modulus β-Ti2448 powder, and TiAl alloy powder showed spherical morphology, compact cross-sections, and no hollow powder defects. Compared with traditional PREP and EIGA processes, they have obvious advantages that are beneficial for promoting the wire plasma atomization technology and the localization of titanium alloy powder. Liu Chang [43] optimized the plasma nozzle structure, designed a piece of supersonic plasma atomization process equipment, and prepared small spherical titanium powder; the powder particles were concentrated in the range of 50 to 74 μm, the powder surface was flat, the surface smoothness and roundness were good, the powder was very close the spherical shape, as shown in Figure 7a, and there were also some satellite powder balls and hollow powder, as shown in Figure 7b, which met the requirements of powder for 3D printing in medical, aviation, and other aspects. The structure of spherical titanium powder prepared by plasma atomization technology is shown in Figure 7.

Metal powder prepared by plasma atomization has a narrow particle size distribution. Powder with a particle size of no more than 53 μm has a high recovery rate, high sphericity, low impurity content, high purity, fine powder particle size, smooth surface, good fluidity, and high atomization efficiency. It is especially suitable for metals with high chemical activity, such as titanium and tungsten. Because the PA technology adopts the atomization method of metal wire to prepare powder, the manufacturing cost of raw materials is high, and the method of metal wire also limits the production efficiency, making it difficult to realize the rapid large-scale production of a single piece of equipment. It also has disadvantages such as expensive equipment, complex processes, high energy consumption, and high cost [44].

The process characteristics and powder size of different milling technologies are summarized in Table 1. PREP technology has the best comprehensive properties of powder, including sphericity, fluidity, and proportion of hollow spheres, and it has obvious advantages in the application of LMD additive manufacturing [45,46]. However, it has high equipment costs and is limited by electrode speed, and the fine-powder harvest rate is low, which limits its further promotion. PA technology has the highest utilization rate of raw materials and a high fine-powder rate, making it the most suitable powder preparation process for SLM additive manufacturing at present. However, it also has some problems, such as complex processes, high operating costs, difficulty in scaling production, expensive equipment prices, and large energy consumption. GA technology has the advantages of high production efficiency, strong process controllability, good repeatability of powder characteristics, controllable fine-powder harvest rate, low production costs, and other advantages. The equipment process is relatively simple, with high repeatability, and the equipment costs and operating costs are relatively low. In particular, EIGA technology effectively avoids pollution in the melting process, shortening the reaction time between the melt and residual oxygen and nitrogen. As a result, the purity of the powder is improved, and it becomes an effective and easy-to-promote industrial atomization pulverization technology for preparing titanium alloy powder.

## 4. The Impact of Process Parameters on the Characteristics of Alloy Powders

### 4.1. Effects of Gas Atomization Process Parameters on the Characteristics of Titanium Alloy Powders

The primary process parameters of gas atomization technology that influence the properties of alloy powder encompass the configuration of the gas nozzle, the structure and placement of the melt delivery pipe, the characteristics of the gas, the atomization pressure, the air velocity, the properties of the metal liquid flow, the degree of superheat, and the diameter of the liquid flow. Among these, the atomization pressure, melting power, and the configuration of the drain pipe are the most critical parameters affecting the characteristics of the powder.

**Figure 8:**
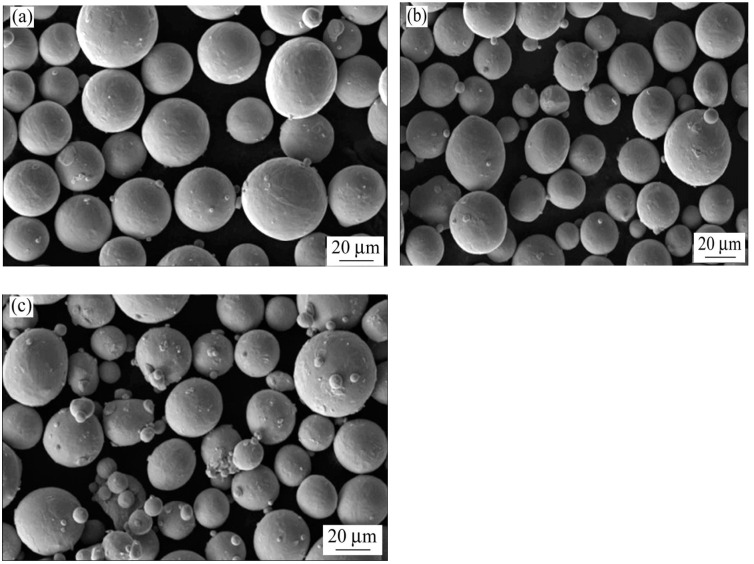
SEM morphologies of TC4 powders prepared at different gas pressures: (**a**) 3.5 MPa, (**b**) 6.0 MPa, (**c**) 7.0 MPa. Reprinted from Ref. [49].

Atomization pressure is a key factor that influences the properties of metal powder and serves as a significant energy source for the atomization medium to fragment the metal liquid and transform gas kinetic energy into surface energy of metal droplets. It directly impacts the particle size distribution and surface morphology of the metal powder. Within a specific pressure range, the particle size of the metal powder tends to decrease as the atomization pressure increases. However, if the atomization pressure continues to rise, the metal liquid may be obstructed at the atomization or diversion nozzles, thereby diminishing the stability and efficiency of the gas atomization process. Guo Kuaiguai [47] employed a high-pressure nozzle gas atomization system to investigate the impact of atomization pressure on the surface morphology, particle size distribution, and flowability of TC4 titanium alloy powder by varying the atomization pressure from 5.0 MPa to 7.0 MPa, while keeping the melting power and the length of the catheter extension constant. The findings indicated that the average particle size of the powder progressively decreased with the increase in gas pressure, reducing from 130.95 μm to 93.66 μm. At lower gas pressures (5.0, 5.5 MPa), the powder particles were thicker, with a certain proportion of irregular, rod-like, and dumbbell-shaped particles. At pressures of 6.0 MPa and 6.5 MPa, the powder exhibited good sphericity and surface finish. When the gas pressure reached 7.0 MPa, due to excessive pressure, the collision and deformation among the powder particles were intensified, resulting in an increase in satellite balls and irregularly shaped particles, and even broken particles, which severely compromised the quality of the powder. Gas pressure at 6.0 MPa and 6.5 MPa is more suitable for laser melting deposition technology. Related studies indicate that higher atomizing gas pressure and airflow velocity increase the interaction between the airflow and metal droplets, leading to greater differences in velocity and solidification state among particles. This, in turn, raises the likelihood of metal droplets colliding with each other, resulting in an increased number of satellite powders and a decline in mobility. Fan Zhao [48] utilized gas atomization to produce TA15 powder for laser selective melting molding and investigated the impact of atomization pressure on the particle size, yield, bulk density, and fluidity of the powder. The findings revealed that as atomization pressure increased, the powder D50 in the 15–53 µm range initially decreased and then increased. Conversely, the bulk density decreased, and fluidity gradually worsened. Additionally, the number of satellite spheres increased, and sphericity was poor. Jin Ying [49] investigated the influence of atomization pressure on Ti-6Al-4V (TC4) alloy powders produced via electrode induction melting gas atomization. The study revealed that within the pressure range of 3.5–6.0 MPa, increasing atomization pressure led to a gradual reduction in mean particle size and an increase in fine-powder yield (<53 μm). At 6.0 MPa, the average particle size reached 48.6 μm with a fine-powder recovery rate of 40.8%. However, further pressure elevation to 7.0 MPa resulted in coarser particles and reduced fine-powder yield. Morphological analysis showed optimal sphericity at 3.5 MPa, while higher pressures induced satellite particles, hollow structures, and non-spherical defects. Both bulk density and flowability decreased with increasing atomization pressure. SEM micrographs of TC4 powders under different atomization pressures are illustrated in Figure 8.

Melting power directly influences the melting speed and temperature of metal droplets. Higher melting power and shorter melting times can enhance melting efficiency, while also increasing the temperature of the metal droplets. Chen Jialu [50] employed EIGA to study the preparation of spherical TC4 alloy powder for laser 3D printing and examined the effects of varying melting power (55–70 kW) on the characteristics of the resulting alloy powder. The study found that with all other factors constant, as EIGA power increased, droplet mass first increased and then decreased. At 60 kW, droplet mass peaked (further increasing power accelerates melting speed, leading to a shortage of rod supply, thereby relatively reducing the effective power involved in melting, thus decreasing alloy droplet mass). The prepared TC4 alloy powder was spherical, with a smooth and clean surface and good sphericity, reaching up to 98%. Guo Kuaiguai’s [51] research showed that increasing smelting power reduced the particle size of TC4 alloy powder and improved powder fluidity. The powder prepared at 56 kW exhibited optimal performance, meeting the requirements of the SLM process. Jiang Bao-lin [52] discovered that as the melting power increased, the superheat of a TA15 titanium alloy solution increased, and the improvement in viscosity gradually raised the powder recovery rate from 26% at 25 kW to 42% at 40 kW.

The drainage tube primarily affects atomization performance by influencing the mass flow rate of the metal liquid. The protrusion length of the drainage tube significantly impacts the gas flow field, directly affecting the stability and efficiency of atomization. Increasing the protrusion length of the drain tube reduces the scope of the reflux zone and shifts the stagnation point downward, creating negative pressure near the end of the drain tube. This exerts a strong suction effect on the metal liquid in the crucible and slightly increases air velocity. Li Xiang [53] found that as the inner diameter of the drainage tube increased, the degree of mutual adhesion among powder particles increased, the tendency to form “satellite balls” in large granular spherical powders grew, and the powder collection rate decreased. The oxygen content (mass fraction) in the powder also showed a significant downward trend. When the inner diameter of the drainage tube’s leakage is less than 4.5 mm, frequent blockages occur, leading to failed atomization. The optimal inner diameter for leakage is 4.5 mm, where the powder recovery rate can exceed 50%, powder sphericity is good, satellite balls are fewer, and the powder meets performance requirements.

### 4.2. Effects of Centrifugal Atomization Process Parameters on the Characteristics of Alloy Powders

The primary process parameters of centrifugal atomization technology that influence the characteristics of alloy powder encompass electrode rod size, rotation speed, cooling gas ratio, feed rate, current intensity, material density, and surface tension [8]. The rotation speed of the electrode rod and the plasma current are critical process parameters affecting the characteristics of the powder.

Tao Yu [54] investigated the impact of process parameters on the average particle size and distribution of produced FGH95 superalloy powder. It was discovered that the powder size distribution correlates with process parameters such as rod speed, plasma arc current intensity, and the distance between the plasma gun and the rod end. Increasing the rod speed, decreasing the plasma arc current intensity, or reducing the distance between the plasma gun and the rod end can narrow the powder size distribution range. By optimizing process parameters like speed, current, and gas flow, the optimal parameters were identified: a rod speed of 14,000 r/min, a plasma arc current of 1200 A, a plasma working gas flow of 80 L/min, and a distance between the plasma gun and rod end of 10 mm, resulting in a high powder recovery rate of 50–100 µm. Li [55] suggested that the initial plasma arc current should be controlled between 800 and 1000 A. Tang [56] examined the characteristics of Ti-6Al-4V powder produced by the plasma rotating electrode process at various speeds. The findings indicate that as speed increases, the average particle size of the powder decreases and the distribution becomes narrower. At higher speeds, there are fewer satellite and irregular particles, as the atomization mode at lower speeds may transition from the liquid line crushing (LD) mode to the direct droplet crushing (DDF) mode [34]. Consequently, empirical formulas are inadequate for predicting particle size at low rotational speeds. Liu Shaowei [57] analyzed the effects of electrode speed and plasma current process parameters on powder properties through experiments. The results demonstrated that as electrode speed increases, centrifugal force rises and powder particle size decreases. With higher current, irregular powder increases, and when the current is excessively large, there is a phenomenon of partial ablation of low-boiling-point elements and the formation of flaky powder. The increase in motor speed can improve the yield of fine powder, but the current equipment maintenance cost is high, and it will also greatly increase the cost of powder, which is far from economical compared with the improvement of fine-powder yield by gas atomization.

### 4.3. Effects of Plasma Atomization Process Parameters on the Characteristics of Alloy Powders

The primary process parameters of plasma atomization technology that influence the characteristics of alloy powder encompass the feed rate of the metal wire, the flow rate of the inert gas, the power of the plasma torch, and the dimensions of the reaction chamber. The optimization of these process parameters is crucial for the preparation of high-quality metal powder using PA technology. Among these, the wire feed rate and the power of the plasma torch are the two most critical variables in the plasma atomization process [58].

The plasma torch serves to transform electrical energy and gas kinetic energy into the thermal energy required to melt the metal wire and the surface energy of the metal powder. Augmenting the power of the plasma torch can enhance the thoroughness of the metal wire’s melting and achieve a higher melting temperature. If the feed rate and inert gas pressure of the metal wire are meticulously controlled, along with the cooling rate, alloy powder with a high sphericity, low oxygen content, and small particle size can be produced [59]. Dai [60] observed that excessive torch power or a low wire feed rate can lead to the melting of the wire occurring above the focus of the plasma jet, resulting in the formation of large molten droplets at the nozzle tip and a decrease in atomization efficiency. On the other hand, a high wire feed rate or low torch power can result in incomplete melting, causing powder agglomeration. The optimal atomization effect is achieved when the wire feed rate is between 10 and 100 g/min and the ratio of wire feed mass to plasma torch power is within the range of 19–32 g/(kW/h).

## 5. Conclusions and Prospects

The quality of powder can be effectively improved by optimizing the process of gas atomization powder production. Specifically, by optimizing the pressure, the yield of fine powder (<53 μm) can be increased by 10~20%, and the energy consumption can be reduced by 20%. By the superheat degree, the yield of fine powder (<53 μm) can be increased by 10~15%. Different processes have different requirements for the particle size of the powder, and the production costs of different types of alloys are also different. Overall, the average production cost of gas atomization powder can be reduced by about 20%.

In the future, the development direction of additive manufacturing technology will exhibit a trend towards diversification, scaling up, and increased intelligence. This evolution brings numerous challenges and opportunities to the research and development of the additive manufacturing of special materials, powder preparation technology, and equipment applications. Achieving high-quality, low-cost, and controllable-particle-size alloy powder remains the primary focus of powder preparation technology. The development of specialized materials for metal 3D printing will continue to be a significant research area for the foreseeable future.

(1) The advancement of new and efficient powder preparation technology and equipment is crucial. Building upon existing powder preparation technologies, further research into the atomization mechanism within metal powder preparation is necessary. It is important to avoid technical defects of various processes, leverage their respective technical strengths, and develop composite powder preparation technology and equipment. This is particularly relevant for the creation of specialized equipment to produce high-end application materials such as titanium alloys, high-purity nickel-based superalloys, alloy steels, and other metal powders. The goal is to produce powders with a low cost, uniform oxygen and nitrogen content, sphericity, a controlled particle size distribution, fluidity, and bulk density, ensuring stable batch quality.

(2) Optimizing process parameters or equipment structure to enhance the yield of target powder is essential. Given the current situation where the optimal target powder (particle size 15–53 μm) recovery rate is only 30–35%, improvements should be made on the basis of existing additive manufacturing powder preparation technologies. By combining the advantages of each process technology and continuously optimizing the equipment structure, the best process parameters can be matched to improve the powder recovery rate.

(3) The development of new alloy powder materials is imperative. In powder design, we should not only consider the strengthening effect but also integrate material composition and process design with machine learning and high-throughput computing. Targeted design and planning of coating structures and cladding strategies should be conducted, establishing a database related to thermophysical and chemical properties. By adjusting the composition, organization, and structure of coatings, the expected performance can be analyzed. Efforts should be made to accelerate the development of wear-resistant coating materials and other functional coatings, in conjunction with laser additive manufacturing control and precision technology, to realize the application of material-structure-performance remanufacturing technology.

## Figures and Tables

**Table 1:** Characteristics of different pulverization technology processes and applicable fields.

Technological Process	Particle Size Range (µm)	Yield (<53 μm)	Sphericity (%)	Oxygen Content (ppm)	Advantages	Disadvantages	AM Applicability
Vacuum induction melting gas atomization (VIGA)	0–180	70%	85–90	200–1400	High production efficiency, strong process controllability, and low production cost	The powder surface is rough, with defects such as connected satellite particles and hollow particles, and it is not possible to prepare reactive powders	SLM/LMD
Electrode induction melting gas atomization (EIGA)	0–180	30–45%	90	1000–1500	Good process reproducibility, higher stability, capable of preparing reactive powders, low production costs	Defects of attached satellite particles and hollow particles	SLM/LMD
Plasma rotating electrode process (PREP)	60–250	≤15%	98	50–800	High sphericity, smooth particle surface, and low oxygen content	Most powder particle sizes are greater than 70 μm, high requirements for equipment	Has obvious advantages in the application of LMD
Plasma atomization (PA)	0–150	32–40%	95	<1000	High purity, fine powder particle size, and stable quality	Low productivity, complicated processes, and high manufacturing costs of wire materials	Most suitable for SLM

## Data Availability

No new data were created or analyzed in this study. Data sharing is not applicable to this article.

## References

[1] Zhang X.J., Tang S.Y., Zhao H.Y., Guo S.Q., Li N., Sun B.B., Chen B.Q. (2016). Research Status and Key Technologies of 3D Printing Technology. J. Mater. Eng..

[2] Li A., Luo C., Yang B.W., Zhang P., Zhao X.H., Lai Y.J. (2024). Research Progress on Titanium Alloys and Powder Preparation Technologies for 3D Printing. Powder Metall. Ind..

[3] Wang H., Peng Y., Zhao L., Tian Z.L. (2021). Research Status and Prospects of Selective Laser Melting 3D Printed TiAl-Based Alloys. Surf. Technol..

[4] Mariani F.E., Ribeiro K.S.B., Lombardi A.N., Srikanth B., Suvin P.S. (2021). Effect of laser polishing post processing technique on the roughness and wear resistance of Inconel 625 deposited by laser cladding on AISI 304L stainless steel. J. Mater. Eng. Perform..

[5] Gu D.D., Zhang H.M., Chen H.Y., Zhang H., Xi L.X. (2020). Laser Additive Manufacturing of High-Performance Metallic Material Components for Aerospace Applications. Chin. J. Lasers.

[6] Yang X.B., Zhu J.L., Chen B.K., Feng Z.H., Li X. (2022). H Research status of plasma rotating electrode atomization technology and powder size control. Powder Metall. Ind..

[7] Tan J.H., Wong W.L.E., Dalgarno K.W. (2017). An overview of powder granulometry on feedstock and part performance in the selective laser melting process. Addit. Manuf..

[8] Hou W.Q., Meng J., Liang J.J., Qiu K.Q., Ren Y.L., Li J.G., Wang D.H., Zhang P., Zhang H.W., Tang G.Q. (2022). Preparation technology and research progress of high-temperature alloy powder for additive manufacturing. Powder Metall. Technol..

[9] Wen J.H., Ding Y.C., Zhao Z.L. (2023). Application Status and Prospects of Additive Repair Technology. Appl. Laser.

[10] Cui X., Zhang S., Zhang C.H., Wu C.L., Wang Q., Dong S.Y. (2020). Research status and prospects of laser additive manufacturing for high-performance functionally graded materials. J. Mater. Eng..

[11] Wang H.M. (2014). Materials′ fundamental issues of laser additive manufacturing for high-performance large metallic components. Acta Aeronaut. Astronaut. Sin..

[12] Yang Q., Lu Z.L., Huang F.X., Li D.C. (2016). Research status and development trends of laser additive manufacturing technology. Aviat. Manuf. Technol..

[13] Xu D., Gao H.B., Dong T., Cui C.Y., Yang Z.L., Li H.X., Jiang C.F., Wang J.D. (2021). Research Progress on Metal Powders for Additive Manufacturing. Chin. J. Nonferrous Met..

[14] Wu Q.Y., Wu Y.J., Deng Q.C., Chang Z.Y., Ding C.Y., Liang Y.Y., Jin Y.H., Han X., Li X.Y., He B.W. (2024). Research Status and Prospects of Additive Manufacturing Technology for Magnesium Alloys and Magnesium-Based Materials. Nonferrous Met. Eng..

[15] Ma J.C., Liu J.D., Zhang Z.P., Kong X.W., Li J.G. (2025). Research status and prospects of laser additive manufacturing of nickel-based superalloys. Met. Process. Hot Work..

[16] Zhang B., Chen S.Y., Liang J., Liu C.S., Cui T., Wang M. (2019). Current status and development trends of laser remanufacturing for short-stress-line rolling mills. J. Mater. Eng..

[17] Zhang K., Yang B.W., Qin L.D., Li K.H., Zuo Z.B., Zhao X.H., Lai Y.J., Liang S.J. (2023). Research Progress in Powder Preparation Technology for Metal Additive Manufacturing. Met. Process. Hot Work.

[18] (2025). Additive Manufacturing of Metals—Environment, Health and Safety.

[19] (2023). High-Entropy Alloy Powder for Additive Manufacturing.

[20] Lawley A. (1992). Atomization: The Production of Metal Powders.

[21] Antony L.V.M., Reddy R.G. (2003). Processes for production of high-purity metal powders. J. Miner..

[22] John H.M. (2000). Utilization of gas-atomized titanium and titanium-aluminide powder. J. Miner..

[23] Zou Y., Liao X.J., Lai Q., Liu Q.C. (2019). Research Status of Preparation Technology of Spherical Titanium Powder for 3D Printing. China Mater. Prog..

[24] Hohmann M., Pleier S. (2015). Production methods and applications for high-quality metal powders and sprayformed products. Acta Metall..

[25] Zhao S.Y., Shen L., Yin J.O., Li Z.F. (2024). Preparation of TiSi_2_ spherical alloy powder by gas atomization and its properties. Powder Metall. Ind..

[26] Zhang G.Q., Zhang Y.W., Zheng L., Peng Z.C. (2019). Research progress on powder superalloys and preparation technology for aero. Acta Metall. Sin..

[27] Wang H., Peng Y., Zhao L., Tian Z.L. (2024). Characterization of Ti-48Al-2Cr-2Nb intermetallicders prepared by electro-induction melting gas atomization metho. Rare Metals.

[28] Hu J.Q., Cheng Z.H., Bai B. (2022). Research Progress in Process Simulation of Atomization Powder Manufacturing. Powder Metall. Ind..

[29] Zai X.F., Chen S.Q., Liu Y., Li R.D., Wu H. (2019). Preparation of Ti6Al4V powder with high yield of fine particle by cru-cible-less gas atomization technology. Rare Met. Mater. Eng..

[30] He X.Y., Li X.G., Huang Y.H., Zhu Q. (2022). Gas flow rectification-based satellite powder control technology in gas atomization powder preparation process. Powder Metall. Technol..

[31] Tang P.J., Ren Z.Z., Yang K.Y., Ma Q., Zhang J., Yang X.Y., Zuang Y.R., Huo W.S. (2023). Research Progress in Preparation Process of 3D Printing Metal Powders by Gas Atomization Method. Mater. Mech. Eng..

[32] Han S.B., Zhang Y.W., Tian X.J., Liu M.D., Jia J. (2017). Research and application of high-quality 3D printing metal powders for aerospace. Powder Metall. Ind..

[33] Gang C., Shao Y.Z., Ping T., Jing O.Y., Quan Z., Yuan G.E., Zeng F.L., Jian W., Hui P.T., Peng C. (2017). Shape memory TiNi powders produced by plasma rotating electrode process for additive manufacturing. Trans. Nonferrous Met. Soc. China.

[34] Tang H.P. (2023). Research Progress in Plasma Rotating Electrode Powder Preparation Technology. Powder Metall. Technol..

[35] Yang X., Xi Z.P., Liu Y., Tang H.P., He W.W., Jia W.P. (2010). Performance characterization of TiAl powder prepared by plasma rotating electrode process. Rare Met. Mater. Eng..

[36] Xie Z.H., Fu A., Wang J., Wang X.F., Cao Y.K., Liu B., Liu Y. (2024). Microstructure and mechanical properties of electron beam selective melting TaNbTiZr refractory high-entropy alloy. Chin. J. Nonferrous Met..

[37] Huber F., Bartels D., Schmidt M. (2021). In situ alloy formation of a WMoTaNbV refractory metal high entropy alloy by laser powder bed fusion (PBF-LB/M). Materials.

[38] Li Z.F., Tan P., Shen L., Zhao S.Y., Wang L.Q., Li A.J., Yin J.O. (2022). Preparation and properties of Ti-Al-8V-5Fe alloy powder. Powder Metall. Technol..

[39] Li B.Q., Jin H.C., Zhang Y.C., Hu P., Yuan F.L., Chen Y.F. (2017). Research Progress in Preparation Technology of Spherical Titanium Powder for 3D Printing. Chin. J. Process Eng..

[40] Tsantrizos P.G., Allaire F., Entezarian M. (1998). Method of Production of Metal and Ceramic Powders by Plasma Atomization. U.S. Patent.

[41] Kroeger I., Marion F. (2011). Raymer AP&C: Leading theway with plasma atomised Ti spherical powders for MIM. Powder Inject. Mould. Int..

[42] Zeng K.L., Luo H., Zhu J., Fu N.K., Lu X.L., Pan C.M., Chen S.S., He P.J. (2021). Research Progress on Titanium Alloy Powder Prepared by Wire Plasma Atomization. Powder Metall. Ind..

[43] Liu C. (2019). Study on Equipment and Process of Plasma Atomization Preparation of Titanium Alloy Powder.

[44] Thompson S.M., Bian L., Shamsei N., Yadollahi A. (2015). An overview of direct Laser deposition for additive manufacturing; Part I: Transport phenomena, modeling and diagnostics. Addit. Manuf..

[45] Chen G., Zhao S.Y., Tan P., Wang J., Xiang C.S., Tang H.P. (2018). A Comparative Study of Ti-6Al-4V Powders for Additive Manufacturing by Gas Atomization, Plasma Rotating Electrode Process and Plasma Atomization. Powder Technol..

[46] Gang R., Yu D.L., Gan L., Ying J., Xiang G.L. (2025). Impact of Atomization Methods on Aluminum Alloy Powder Characteristics and 3D Printing Performance in Laser Directed Energy Deposition Process. Trans. Nonferrous Met. Soc. China.

[47] Guo K.K., Shang S., Chen J., Chen J., Liu C.S. (2020). Numerical simulation of the effect of atomization gas pressure on particle size of GH4169 alloy powder. J. Northeast. Univ. Nat. Sci..

[48] Fan Z., Cheng H., Yuan C.F., Liu Q.W. (2022). Preparation of TA15 alloy powder and its SLM forming performance. Ordnance Mater. Sci. Eng..

[49] Jin Y., Liu P., Shi J.G., Weng Z.Q., Gu X.L. (2018). Effect of atomization pressure on morphology and properties of TC4 powder prepared by electrode induction melting gas atomization. Mater. Sci. Eng. Powder Metall..

[50] Chen J.L., An R., Dong H.H., Zhang L. (2023). Effect of EIGA Process Parameters on the Characteristics of TC4 Alloy Powder for Laser 3D Printing. Powder Metall. Ind..

[51] Guo K.K., Liu C.S., Chen S.Y., Fu Q. (2017). Effect of Power on Characteristics of TC4 Alloy Powder Prepared by EIGA for 3D Printing. Mater. Sci. Technol..

[52] Jiang B.L., Guan X.Y., Ye G.C., Jiang C. (2024). Effects of Pressure and Power on the Preparation of TA15 Powder by EIGA. Equip. Manuf. Technol..

[53] Li X., Zeng K.L., He P.J., Luo H., Zhu J., Song X.Q. (2021). Effect of close-coupled gas atomization parameters on properties of metal powders for 3D printing. Powder Metall. Technol..

[54] Tao Y., Di F., Zhang Y.W., Guo W.M., Zhang Y., Zhang F.G., Chen S.D. (2003). Effect of PREP Process Parameters on Powder Characteristics of FGH95 Superalloy. J. Iron Steel Res..

[55] Li L., Dai Y.U., Lv P. (2018). Study on the Preparation Process of AlSi10Mg Aluminum Alloy Powder by Plasma Rotating Electrode Method. New Mater. Ind..

[56] Tang J.J., Nie Y., Lei Q., Li Y.P. (2019). Characteristics and atomization behavior of Ti-6Al-4Vpowder produced by plasma rotating electrode process. Adv. Powder Technol..

[57] Liu S.W., Duan W.C., Dong B.B. (2019). Effect of PREP process parameters on the properties of AlSi10Mg aluminum alloy powder for 3D printing. Nonferrous Met. Eng..

[58] Abhishek M., Thinh H., Nemanja K., Kevin G., Asif M., Marko K., Brandon M., Kyu C., Yongho S. (2023). Additive Manufacturing of Al18Co30Cr10Fe10Ni32 High Entropy Alloy by Gas Atomization and Laser Powder Bed Fusion. Mater. Lett..

[59] Wu W.H., Wang T., Fan D. (2021). Research Progress on Key Preparation Technologies of Spherical Metal Powders for Additive Manufacturing. Mater. Mech. Eng..

[60] Dai Y., Li L. (2018). Analysis of Plasma Torch Atomization Technology for Preparing Titanium Alloy Powder Specialized in Metal 3D Printing. New Mater. Ind..

